# mHealth-Augmented Care for Reducing Depression Symptom Severity Among Patients With Chronic Pain: Exploratory, Retrospective Cohort Study

**DOI:** 10.2196/52764

**Published:** 2025-01-10

**Authors:** Dan Holley, Amanda Brooks, Matthew Hartz, Sudhir Rao, Thomas Zaubler

**Affiliations:** 1 Clinical Operations, NeuroFlow, Inc, 1601 Market St, Suite 1500, Philadelphia, PA, 19103, United States, 1 3026893520; 2 Data and Engineering, NeuroFlow, Inc, Philadelphia, PA, United States; 3 Pain and Spine Specialists, Mt. Airy, MD, United States

**Keywords:** mHealth, mobile health, app, behavioral health care, depression, mental health, screening, pain, chronic pain, psychiatric screenings, digital health care, psychiatry, psychiatric, longitudinal, assessment, behavioral, self-help, integrated, comorbidity, augmented care

## Abstract

**Background:**

Depression and chronic pain are commonly comorbid, mutually reinforcing, and debilitating. Emerging approaches to mobile behavioral health care (mHealth) promise to improve outcomes for patients with comorbid depression and chronic pain by integrating with existing care models to bolster support and continuity between clinical visits; however, the evidence base supporting the use of mHealth to augment care for this patient population is limited.

**Objective:**

To develop an evidence base that sets the stage for future research, we aimed to explore the associations between changes in depression severity and various integrated care models, with and without mHealth augmentation, among patients with comorbid depression and nonmalignant chronic pain.

**Methods:**

Our team leveraged retrospective, real-world data from 3837 patients with comorbid depression and nonmalignant chronic pain who received integrated behavioral health care (IBH) at a subspecialty pain clinic. We analyzed one IBH-only, non-mHealth cohort (n=2765), an mHealth-augmented cohort (n=844), and a collaborative care (CoCM)+mHealth cohort (n=136), which were supported by the NeuroFlow mHealth platform, and a pre-CoCM mHealth cohort (n=92), which was supported by the mHealth platform for 3 months prior to beginning the chronic pain treatment. We evaluated changes in depression severity between treatment cohorts via longitudinal analyses of both clinician- and mHealth-administered Patient Health Questionnaire-9 (PHQ-9) assessments.

**Results:**

mHealth-augmented integrated care led to significantly greater proportions of patients reaching clinical benchmarks for reduction (725/844, 86% vs 2112/2765, 76%), response (689/844, 82% vs 2027/2765, 73%), and remission (629/844, 75% vs 1919/2765, 69%) compared with integrated care alone. Furthermore, hierarchical regression modeling revealed that patients who received mHealth-augmented psychiatric CoCM experienced the greatest sustained reductions in on-average depression severity compared with other cohorts, irrespective of clinical benchmarks. In addition, patients who engaged with an mHealth platform before entering CoCM experienced a 7.2% reduction in average depression severity before starting CoCM treatment.

**Conclusions:**

Our findings suggest that mHealth platforms have the potential to improve treatment outcomes for patients with comorbid chronic pain and depression by providing remote measurement–based care, tailored interventions, and improved continuity between appointments. Moreover, our study set the stage for further research, including randomized controlled trials to evaluate causal relationships between mHealth engagement and treatment outcomes in integrated care settings.

## Introduction

America’s psychiatric crisis impairs our health, happiness, and productivity on an industrial scale. Half of all Americans will experience a behavioral health condition at some point in life, and 1 in 5 will experience a condition so severe that it is debilitating [1,2]. Despite the ubiquitous nature of these conditions and their sprawling impacts, psychiatric care is underused, and many individuals resist treatment up to the point of emergency crises [3,4].

Exacerbating this, behavioral health conditions are frequently accompanied by physical health complications. For instance, depression and chronic pain are commonly comorbid [5-10] and feature prominently atop leading causes of disability worldwide [1]. Along with 23% to 37% higher annual health care costs [11], individuals who live with chronic pain experience increased risk of suicidality [12,13], reduced quality of life [14-16], poor occupational fulfillment [17], and disrupted family dynamics [18,19]. While chronic pain’s causal contributions to depression are intuitive and well-established, emerging neuroscientific research indicates that this relationship is bidirectional: depression can sensitize the body to pain via multiple mechanisms across a shared neural substrate [20-22]. Thus, treating either depression or chronic pain alone may be less effective than treating both conditions in an integrated care setting. Despite the biobehavioral complexities of comorbid depression and pain, treatments are often siloed, which contributes to second-order crises. The opioid epidemic, for example, is enabled by a longstanding reliance on pharmacological treatment for chronic pain [23-25].

Traditional treatment models that focus on either behavioral or physical health conditions in isolation often fail to address the complex interplay between comorbid conditions like depression and chronic pain. In the absence of integrated care approaches, patients face fragmented care pathways, which can exacerbate symptoms, lead to suboptimal outcomes, and contribute to treatment resistance [5,23,26,27]. Without mHealth augmentation, health care providers are limited in their ability to monitor patients between visits, track real-time changes in health status, and intervene before crises occur [28-31]. These challenges are particularly pronounced in patients with chronic pain and comorbid depression, who may be at higher risk of falling through the gaps of episodic care models.

Integrated treatment models, notably psychiatric collaborative care (CoCM), have emerged as sophisticated approaches in the treatment of complex patients who present with behavioral and physical health comorbidities [26,27,32]. Under these models, multifaceted care teams provide integrated, population-based, patient-centered behavioral and physical health care. In CoCM, for example, medical providers, behavioral health care managers, caseload supervising psychiatrists, and patients share records and work together as one team to establish and achieve goals, thereby enabling the coordinated treatment of comorbid behavioral and physical health issues, such as depression and chronic pain. While integrated approaches are not widely used, an extensive evidence base attests to their effectiveness and efficiency [26,27,33,34].

Nevertheless, questions remain about whether and how emerging technologies, such as mobile behavioral health care (mHealth) platforms [3,35,36], can augment and scale integrated care models. Despite the surge in the implementation and broadening of mHealth during the pandemic, the utilization of such technologies is still not at its fullest. Stand-alone self-management tools, such as mental health apps, offer limited proven benefits. However, technology-driven solutions designed to complement and expand the mental health workforce hold boundless promise [28]. mHealth is an especially attractive facet of integrated care that promises to improve personalized treatment and enhance patient continuity via remote measurement–based care and tailored, real-time digital interventions. Critically, mHealth platforms can provide scalable, location-agnostic support that reaches patients where they are, when they need it, thereby helping to overcome barriers, such as provider scarcity and treatment stigma [29-31]. They also help to elevate and standardize the quality of behavioral care by providing clinical decision support for providers and digital self-management tools for patients, all of which facilitate compliance with evidence-based care.

While much has been written about the advantages of mHealth [28-31,35,36], our study is among the first to empirically investigate its use among integrated behavioral health care (IBH) patients with comorbid chronic pain and depression, in a real-world setting. To better understand the relationships between mHealth-augmented treatment approaches and patient outcomes in cases of comorbid behavioral and physical health conditions, we conducted an exploratory, retrospective cohort study of behavioral health outcomes in 3837 patients who received IBH at a subspecialty pain clinic. We analyzed data collected during routine health care operations to evaluate changes in depressive symptomatology over time among cohorts defined by the type of IBH administered and the availability and timing of mHealth support. Our objective was to evaluate the associations between mHealth support and behavioral health outcomes among patients with chronic pain, and to provide new insights into the potential for mHealth to augment IBH.

## Methods

### Study Design

We used a retrospective-cohort design to explore the associations between treatment approaches and changes in depression severity among 3837 patients aged 18 years and over who showed signs of clinical depression and received treatment for chronic nonmalignant pain at a subspecialty pain clinic between April 2019 and April 2023.

### Patient Cohorts

To understand the links between different treatment approaches and changes in depression severity, we organized our retrospective patient population into 4 distinct treatment cohorts, further described in Table 1. Because this was a retrospective study of real-world data, patients were not assigned to cohorts, and we have no insight into the process by which patients arrived in their cohorts.

**Table 1.** Treatment cohorts.

		Cohort				
		IBH^a^ (n=2765)	IBH+mHealth^b^ (n=844)	CoCM^c^+mHealth (n=136)	Pre-CoCM mHealth (n=92)	Total: all cohorts (N=3837)
**Sex, n (%)**						
	Female	1824 (66)	610 (72.3)	102 (75)	67 (72.8)	2603 (67.9)
	Male	940 (34)	234 (27.7)	34 (25)	25 (27.2)	1233 (32.1)
Age (years), mean (SD)		55.95 (13.61)	55.60 (13.81)	60.25 (13.06)	50.39 (13.47)	55.71 (13.73)
Treatment description		Patients were regularly evaluated for symptoms of depression using the PHQ-9^d^ during scheduled office visits. Elevated PHQ-9 assessments were reviewed by either an MD or BH^e^ specialist. All patients were provided with stress reduction techniques, including meditation, mindfulness, and deep breathing exercises. Stress-reduction interventions were provided by either an MD or on-site BH specialist.	Patients received IBH as described above, augmented by use of the mHealth app.	Patients received highly integrated medical and BH care under the CoCM, with access to the mHealth app at the start of CoCM care.	A unique group of CoCM patients who had at least 3 months of advance access to the mHealth app prior to the start of CoCM treatment, and who completed at least one mHealth activity per month in those 3 months.	—^f^
Notes		—	—	Because all CoCM patients’ treatment was augmented by mHealth, our study design lacks a CoCM-without-mHealth group for comparison.	—	Between-group age differences were not statistically significant (one-way ANOVA: *F*_3,3833_=1.23; *P*=.30).

a IBH: integrated behavioral health care.

b mHealth: mobile behavioral health care

c CoCM: collaborative care.

d PHQ-9: Patient Health Questionnaire-9.

e BH: behavioral health.

f Not available.

### Measuring Depression Severity

To measure changes in depression severity over time, we evaluated real-world data from clinic- and mobile-administered Patient Health Questionnaire-9 (PHQ-9) assessments [37]. The PHQ-9 is a well-validated and reliable measure for the detection and severity stratification of depressive disorders and is regarded as a suitable tool for clinical depression research. Its 9 questions address the *Diagnostic and Statistical Manual of Mental Disorders, Fifth Edition, Text Revision* components of major depressive disorder on a 0 (“not at all”) to 3 (“nearly every day”) scale. Scores range from 0 to 27, with the following ranges commonly regarded as qualitative differences in depression severity: 0‐4, not depressed; 5‐9, mild depression; 10‐14, moderate depression; 15‐19, moderate-to-severe depression; and 20‐27, severe depression.

### Mobile Behavioral Health Platform

Our study used the NeuroFlow digital behavioral health platform (NeuroFlow, Inc.) to document, monitor, and share updates on patients’ emotional state, sleep patterns, and stress levels, and to perform routine assessments, including the PHQ-9. In addition, the app offers informative videos tailored to users’ self-reported scores and evolving symptoms. Health care providers use the mHealth platform to enhance continuity between visits and track patient-reported outcomes to garner unique insights into the patients’ physical and behavioral well-being.

### Statistical and Computational Methods

To compare initial depression severity of our cohorts, we first established a clinical index for each patient based on PHQ-9 scores. Consistent with Kroenke et al [37], we defined our clinical index as the first occurrence of a PHQ-9 score of ≥10, which indicates moderate (or worse) depression. We then used a one-way ANOVA and subsequent post hoc tests to evaluate our cohorts for mean differences in initial severity. We also computed a one-way ANOVA to test for significant differences in our cohorts’ mean ages at the outset of the study.

We used a series of chi-square tests to compare the IBH and IBH+mHealth cohorts based on the proportion of patients reaching established clinical benchmarks for reduction (ie, a ≥5-point drop in PHQ-9 score recorded any time after clinical indexing), response (ie, a ≥50% drop following clinical indexing), and remission (ie, a score of <5 following clinical indexing). Furthermore, we computed a series of independent-samples *t* tests (2-tailed) to explore differences in the speeds with which each cohort reached these benchmarks.

In addition, we leveraged hierarchical (mixed-effects) regression modeling to examine the relationships between different treatment approaches and the temporal dynamics of depression severity from the time of clinical indexing onward, irrespective of clinical benchmarks. This model accounts for repeated measurements of PHQ-9 scores over time within each patient, treating patients as random effects and treatment cohorts as fixed effects. We calculated the slope and standard error of evolving PHQ-9 scores for each patient and incorporated time as a continuous variable to assess changes in depression severity over time. The model included the fixed effect of cohort, with the IBH, IBH+mHealth, and CoCM+mHealth cohorts treated as categorical variables using dummy coding. We included patient-specific random intercepts and slopes to account for individual variability in depression severity trajectories.

We instantiated this model using the statsmodels library’s *mixedlm()* function in Python 3.8.3. The analysis was implemented as a mixed-effects linear regression with the formula PHQ_SCORE ~ TIME + C(COHORT), where PHQ_SCORE was the dependent variable, TIME represented the temporal evolution of scores, and C(COHORT) specified the treatment cohorts as categorical predictors. We also included the interaction term between time and cohort to evaluate whether changes in PHQ-9 scores over time differed across cohorts. Random effects for both the intercept and the slope of time were included to account for the nested structure of the data, with repeated measures for each patient. This approach allowed us to account for both within- and between-subject variance. The random-effects structure assumed a normal distribution for both random intercepts and slopes, and we fit the model using the Restricted Maximum Likelihood estimation method, ensuring convergence by using the Broyden-Fletcher-Goldfarb-Shanno optimization algorithm.

Other model assumptions included:

1. Random effects for patients: we assumed that individual patients had their own baseline PHQ-9 scores and their own trajectories of change over time.
2. Fixed effects for treatment cohorts: the treatment cohorts were treated as fixed, meaning we expected consistent effects of each cohort across patients.
3. Normality of residuals: we assumed that the residuals of the model followed a normal distribution, and that the relationship between time and PHQ-9 scores was linear.
4. Time as a continuous variable: we assumed that changes in depression severity occurred continuously over time, rather than at specific time points.

Finally, we performed a paired-samples *t* test (2-tailed) to compare mean differences in depression severity among pre-CoCM mHealth patients at two time points: PHQ-9–derived clinical index scores recorded before entering CoCM, and PHQ-9 scores associated with the start of CoCM treatment.

Because our analyses were exploratory, all hypothesis tests were 2-tailed and thresholded at *P*<.05. Results reported here are uncorrected for multiple comparisons. All statistical tests were performed in Python v3.8.3 (Python Software Foundation) using standard statistical libraries (eg, scipy.stats v1.10.1) and functions (eg, *stats.ttest_rel()* for paired-sample *t* tests, *stats.f_oneway()* for one-way ANOVA, etc).

### Organizations Involved

Pain and Spine Specialists is a pain subspecialty clinic with locations in Maryland, Pennsylvania, and Virginia. Pain and Spine Specialists’ network of board-certified clinicians provides chronic pain management and interventional pain treatment in integrated care settings. NeuroFlow, Inc., is a private, for-profit digital behavioral health company headquartered in Philadelphia, Pennsylvania. Its mHealth platform is a Health Insurance Portability and Accountability Act (HIPAA)–compliant, US Food and Drug Administration (FDA)–exempt, cloud-based tool that facilitates behavioral health care access, engagement, and remote measurement–based care.

### Ethical Considerations

All patient data were collected as a facet of routine health care operations and were anonymized prior to analysis. Our study was exempted from institutional review board oversight by Advarra’s Center for IRB Intelligence (protocol number 00069060, dated January 23, 2023), which covered both the collection of data and the waiver of informed consent for our analyses. Data are stored on access-controlled, firewall-protected servers within NeuroFlow, Inc.

## Results

### Comparing Initial Depression Symptoms Between Cohorts

**Figure 1.**
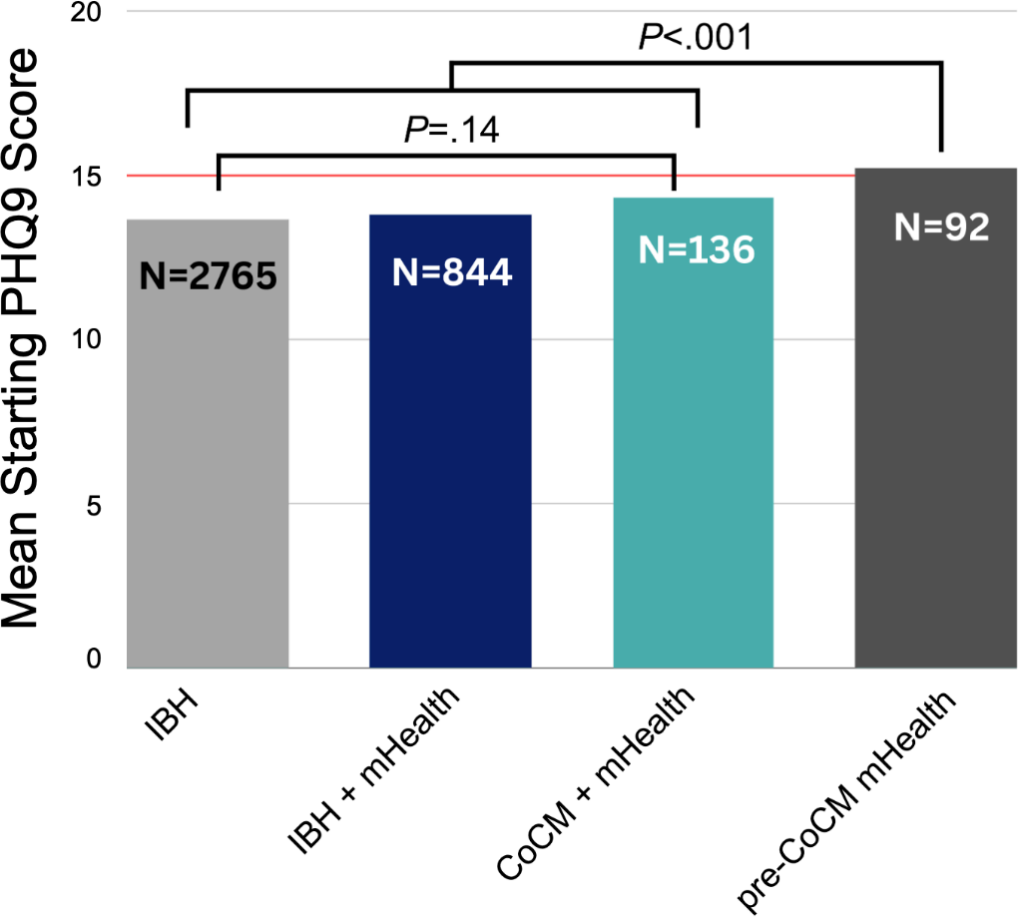
Measuring mean starting depression severity revealed no significant differences by cohort, except for significantly elevated initial depression severity in the pre-CoCM mHealth cohort. The red line illustrates the categorical cutoff for moderate-to-severe depression. This finding informed our downstream analyses by checking against a priori differences between cohorts and resulted in our exclusion of the pre-CoCM mHealth cohort from subsequent groupwise analyses. CoCM: collaborative care; IBH: integrated behavioral health care; mHealth: mobile behavioral health care; PHQ-9: Patient Health Questionnaire-9.

As a first step in evaluating our cohorts, we compared each group’s mean clinical indices to determine whether starting depression severity significantly differed across all groups. Although we expected no significant differences by cohort, that was not always the case (Figure 1). We found the average starting severity for the IBH (13.64, SD 3.91), IBH+mHealth (13.78, SD 3.70), and CoCM+mHealth (14.32, SD 3.69) cohorts to be both categorically (ie, “moderate depression”) and statistically similar (one-way ANOVA, *F*_2,3742_=1.97; *P*=.14). However, average starting severity for the pre-CoCM+mHealth cohort (15.20, SD 4.16) was both categorically (moderate-to-severe depression) and statistically higher than the other cohorts (*F*_3,3833_=5.77; *P*<.001). Given the significant difference in baseline severity, the variability in pretreatment mHealth engagement duration, and the smaller sample size within this cohort, we have excluded it from subsequent between-group analyses to avoid confounding the results.

### IBH+mHealth Is Linked to Proportionately Greater Improvements Than IBH on Its Own

**Figure 2.**
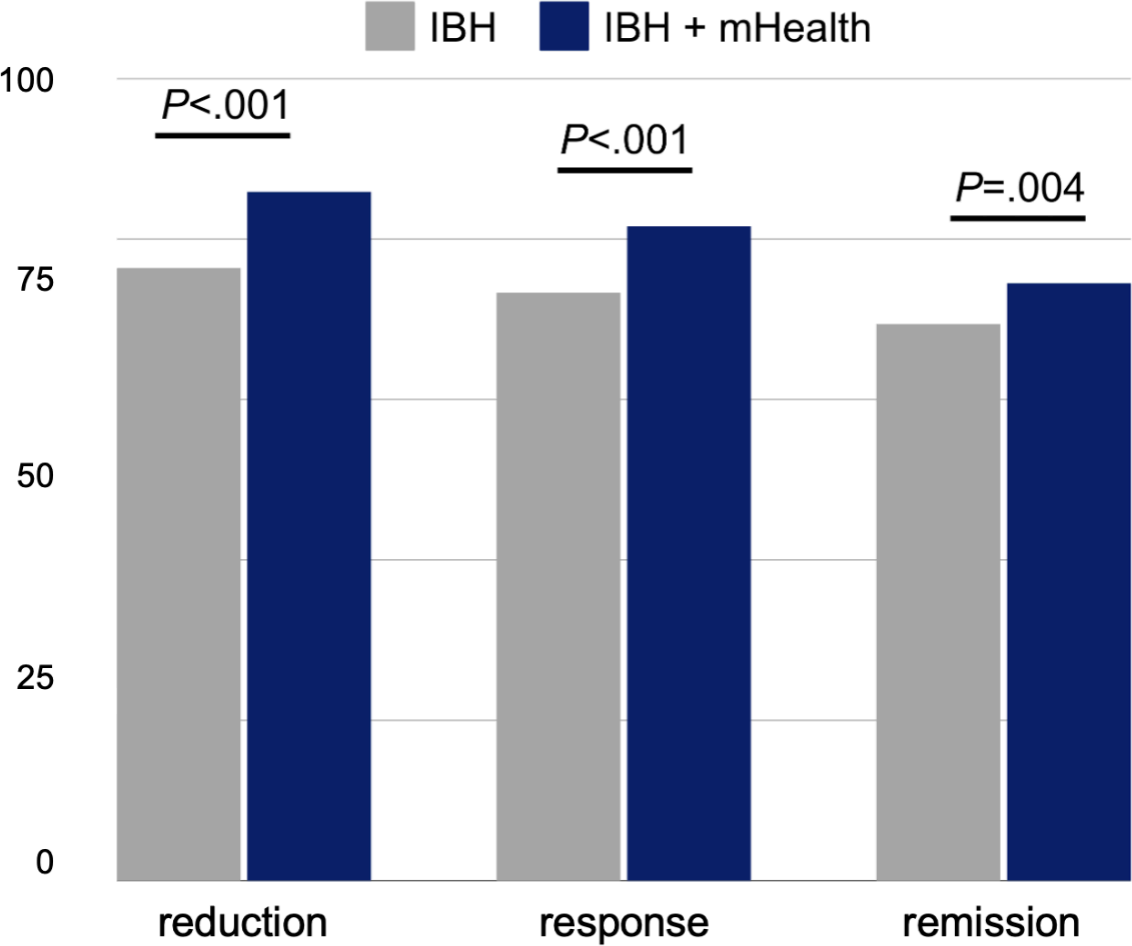
We compared the percentage of patients that reached clinical benchmarks for reduction, response, and remission between our two largest cohorts to provide insight into the links between mHealth augmentation and enhanced patient outcomes. Integrated behavioral health care+mHealth (n=844) was associated with significantly greater proportions of patients reaching clinical benchmarks of reduction (*χ*_1_^2^=111.45; *P*<.001), response (*χ*_1_^2^=28.82; *P*<.001), and remission (*χ*_1_^2^=8.04; *P*=.004) compared with IBH on its own (n=2765). IBH: integrated behavioral health care; mHealth: mobile behavioral health care.

Next, we aimed to dissociate the contribution of mHealth to relationships between treatment types and patient outcomes. To do this, we analyzed the IBH (n=2765) and IBH+mHealth (n=844) cohorts. Patients in these groups received equivalent treatment, with one important exception: the latter also received mHealth-administered content, measurement, and support (see *Methods*). Having already established that these cohorts did not significantly differ in their starting depression severity, we proceeded to test the groups for differences in the proportion of patients who reached the clinical benchmarks for reduction, response, and remission (see Methods). We found that significantly more patients in the IBH+mHealth cohort reached reduction, response, and remission compared with patients engaged in IBH without mHealth support (Figure 2; reduction: *χ*_1_^2^=111.45, *P*<.001; response: *χ*_1_^2^=28.82, *P*<.001; remission: *χ*_1_^2^=8.04, *P*=.004). In total, 85.9% (725/844), 81.6% (689/844), and 74.5% (629/844) of IBH+mHealth patients reached reduction, response, and remission, respectively, compared with 76.4% (2112/2765), 73.3% (2027/2765), and 69.4% (1919/2765) of IBH patients. Surprisingly, however, IBH without mHealth was associated with significantly faster mean times to response (116 d vs 96.1 d; independent-samples *t* test: *t*_2714_=2.08; *P*=.04) and remission (134.9 d vs 113.5 d; *t*_2546_=6.32; *P*<.001) benchmarks compared with integrated care+mHealth. Subsequent hierarchical regression modeling (described below) suggests that this finding may be a byproduct of day-to-day fluctuations in PHQ-9 scoring, and less suggestive of treatment effectiveness, per se. In addition, this finding may reflect the tendency of patients to be more forthright in virtually administered PHQ-9 screenings, which afford greater privacy than in-person screenings [38]. Nevertheless, this finding warrants additional research.

### mHealth-Augmented CoCM Is Linked to Robust Decreases in Depression Severity, Irrespective of Clinical Benchmarks

Although analyzing clinical benchmarks of reduction, response, and remission can be an informative measure of patient outcomes, additional approaches can be useful to account for day-to-day variation in symptoms. PHQ-9 scores fluctuate, so examining overall trends in symptomatology, including scores recorded after reaching a clinical benchmark, provides a more complete picture of patients’ changing depression severity. Because of this, we sought to measure severity trends in each cohort, irrespective of when or whether patients reached clinical benchmarks. To do this, we built a hierarchical regression model with fixed effects of cohort and random effects of individual patients to evaluate the linear associations between treatment approaches and changes in depression severity over time. We calculated within-group regression coefficients and standard errors for each patient and collapsed those values by cohort to compare linear trends between groups using the approach described by Aiken and West [39]. While depression severity among all cohorts improved over time, we found that the CoCM+mHealth cohort was associated with significantly faster, sustained symptom reduction than the IBH (z=2.47; *P*=.01) or IBH+mHealth (z=4.43; *P*<.001) cohort. In CoCM+mHealth, every 8.7 days of care was associated with a sustained, groupwise 1-point decrease in average PHQ-9 score. By comparison, in the IBH cohort, a 1-point decrease occurred every 19.8 days, on average, and, in the IBH+mHealth cohort, this association was 1 point every 43.5 days. Despite the clinically significant difference in trajectories between the IBH and IBH+mHealth cohorts, these groups’ differences were not statistically significant (z=1.25; *P*=.21). Like the clinical benchmark analyses discussed in the preceding section, this finding should be interpreted cautiously in light of established differences between in-person and digitally administered PHQ-9 scores [38].

### Pre-CoCM mHealth Is Associated With Significant Improvements in Depression Severity

A subsection of 92 of our patients had early access to the mHealth app and engaged with mHealth modules at least once per month in the 3 months preceding CoCM treatment. As shown in Figure 1, these patients, on average, had the most severe starting depression of the cohorts we examined, and comprised the only cohort that started with “moderate-to-severe” depression. We hypothesized that this group would see reduced depression severity prior to the start of CoCM. To test this hypothesis, we compared differences in mean clinical index scores (ie, the patients’ first ever PHQ-9 scores of ≥10) to their CoCM index scores (ie, PHQ-9 scores of ≥10 circa commencement of CoCM treatment). We found that pre-CoCM mHealth engagement was associated with a statistically significant reduction in PHQ-9 scores, which fell 7.20% from an average of 15.20 at the time of clinical indexing to 14.10 circa the start of CoCM (paired-samples *t* test: *t*=2.44, *P*=.0). This reduction brought the pre-CoCM cohort from an average starting severity of “moderate-to-severe” to “moderate,” which was then similar to that of the other groups.

## Discussion

### Primary Findings

Here, we have presented the results of an exploratory, retrospective cohort study of real-world data collected from 3837 patients experiencing depression and receiving various levels of integrated care, with and without mHealth support, at a subspecialty pain clinic. While much of the existing literature has focused on the theoretical benefits of mHealth [28,36], our study is one of the first to empirically examine its impact within a real-world integrated care setting among patients with comorbid chronic pain and depression. Our findings contribute to the growing literature on the potential for mHealth to support complex patients between clinical visits, for instance, through the administration of remote assessments [3,35,40]. Moreover, this study provides new evidence linking mHealth support to enhanced behavioral health outcomes, underscoring the need for further research to establish causality and optimize its clinical implementation.

Augmenting integrated care with mHealth technology offers several benefits, including remote automated measurement, real-time digital support, and enhanced engagement between visits. In addition, mHealth-augmented approaches empower providers with nonpharmacological treatment options that could potentially reduce opioid prescribing and, by extension, misuse [41]. Our study suggests that mHealth support can be a valuable tool to reduce depression symptoms in patients who suffer from comorbid chronic pain and depression. Indeed, our CoCM+mHealth cohort saw the most rapid and robust improvements in depression severity, irrespective of clinical benchmarks. While further research is needed, these findings add to the literature evidencing CoCM’s effectiveness [26,27] while hinting at the intriguing possibility that mHealth-augmented CoCM may outperform CoCM on its own. Meanwhile, between the two cohorts in which mHealth was dissociable (ie, IBH and IBH+mHealth, which together comprised 94% of our total sample; Figure 2), a significantly greater proportion of patients achieved clinical reduction, response, and remission when their integrated care was supplemented by the mHealth app. We additionally found that mHealth engagement was associated with a statistically significant decrease in depression severity within a cross-section of CoCM patients who had early access to the mHealth platform.

### Interpreting Our Findings in the Context of the Existing Literature

Our study adds to the growing evidence that mHealth interventions can improve depression outcomes, particularly in integrated care settings. Similar to Kim and Lee [36], who demonstrated that mHealth tools effectively managed depression in real-world populations, we found that mHealth support enhanced outcomes over time. Likewise, Torous et al [28] highlighted the critical role of mHealth in expanding access to mental health care during the COVID-19 pandemic. Importantly, our study suggests that mHealth is more effective when used as part of a coordinated care framework, rather than as a stand-alone tool. This finding aligns with Unützer et al [26], who emphasized that CoCM models are essential for managing patients with comorbid behavioral and physical health conditions, such as depression and chronic pain. Recent studies [42,43] have further confirmed that mHealth’s impact on mental health outcomes is maximized when integrated with comprehensive care systems, supporting our approach.

In contrast to the robust effects seen with integrated mHealth approaches, challenges around patient engagement remain a significant barrier: promoting adherence to mHealth interventions is an ongoing challenge [40] and can be especially difficult among patients with multiple comorbidities [44,45]. This may have been reflected in our pre-CoCM cohort, where variability in engagement potentially influenced the effectiveness of mHealth support (although we hesitate to strongly interpret this cohort’s outcomes; see Results for rationale). While our findings echo the benefits reported in studies like Ye et al’s [46] meta-analysis on internet-based cognitive behavioral therapy, they also underscore the complexities of delivering mHealth interventions in real-world clinical settings, particularly among patients with multiple comorbidities, and reinforce the need for tailored engagement strategies.

### Study Limitations

Several limitations should be noted when interpreting our results. First, our retrospective cohort study lacked a true control group, and we were therefore unable to compare our treatment cohorts to placebo or untreated controls. Because of this, our study was not designed to investigate the potential adverse impact of untreated depression over time, which may be especially relevant for patients with chronic pain. Future prospective cohort studies that do so would help contextualize our findings. We also lacked access to robust electronic health record data, including nondepression comorbidities and social determinants of health–related information, which could have affected our observations. However, the large sample sizes in the IBH and IBH+mHealth cohorts and the a posteriori randomization of our retrospective design likely mitigated the impact of these unmeasured confounds. Furthermore, because we collected PHQ-9 scores but not self-reported pain severity, we are unable to evaluate the associations between treatment cohorts and changing pain symptomatology; nor did we have information on prescription pain medication or opioid use. Finally, a majority (86%) of patients declined to provide optional demographic data (eg, race and ethnicity) during patient intake, which precludes the examination of demographic relationships to outcomes. Nevertheless, our study provides early evidence on the associations between mHealth-augmented integrated care and patient outcomes within a large sample of complex patients.

### Future Directions

Our findings set the stage for future research into the causal effects of mHealth engagement and its potential to augment treatment across the integrated care continuum based on the needs, motivations, and readiness of the patient and their health care system. Patients who present with depression and comorbid chronic pain are ideally suited for future randomized controlled studies examining the impact of mHealth interventions on complex patient outcomes in CoCM settings. Such studies would provide key insights into the potential for evidence-based, depression-centered mHealth content (eg, digital cognitive behavioral therapy modules [46-48]) and remote measurements to reduce physical pain symptoms by targeting one pillar of these mutually reinforcing ailments [6-9,22].

Future research should also explore how mHealth interventions can be systematically integrated into existing care models such as CoCM, primary care, and specialty care settings. Process studies will be crucial in identifying the operational challenges associated with embedding mHealth tools into standard care workflows, such as aligning mHealth use with clinical visits, ensuring data interoperability across platforms, and training care teams to use mHealth insights effectively. These studies could reveal barriers to implementation, such as limited clinician buy-in, technology literacy issues among patients, and regulatory or privacy concerns—any of which may impact the scalability of mHealth solutions. Understanding how mHealth can complement, rather than disrupt, existing workflows will be critical to successful integration.

In parallel, future research should focus on understanding how specific components of mHealth, such as tailored intervention timing and personalized engagement strategies, influence outcomes across different patient populations. Investigating the optimal duration and frequency of mHealth augmentation will also help to establish the minimum effective dose for improving both depression and pain-related outcomes. Studies evaluating long-term outcomes, such as sustained symptom reduction and quality-of-life improvements, would offer valuable data on the durability of mHealth effects.

In addition, cost-of-care analyses should be conducted to explore how mHealth integrates with existing care frameworks and whether it offers cost-effective solutions for managing depression and chronic pain [27,33,34]. Another key area of research is the potential for mHealth to reduce opioid prescribing and misuse, particularly in patients with chronic pain and psychiatric comorbidities [24,41]. Investigating the relationship between mHealth usage, opioid reduction, and substance use disorder prevention could have profound implications for public health policy and pain management strategies.

## Abbreviations

BH: behavioral health
CoCM: collaborative care
FDA: US Food and Drug Administration
HIPAA: Health Insurance Portability and Accountability Act
IBH: integrated behavioral health care
mHealth: mobile behavioral health care
PHQ-9: Patient Health Questionnaire-9
